# smFRET Detection of Cis and Trans DNA Interactions by the BfiI Restriction Endonuclease

**DOI:** 10.1021/acs.jpcb.3c03269

**Published:** 2023-07-15

**Authors:** Šaru̅nė Ivanovaitė, Justė Paksaitė, Aurimas Kopu̅stas, Giedrė Karzaitė, Danielis Rutkauskas, Arunas Silanskas, Giedrius Sasnauskas, Mindaugas Zaremba, Stephen K. Jones, Marijonas Tutkus

**Affiliations:** † Department of Molecular Compound Physics, Center for Physical Sciences and Technology, Savanorių 231, Vilnius LT-02300, Lithuania; ‡ Vilnius University, Life Sciences Center, Institute of Biotechnology, Saulėtekio av. 7, Vilnius LT-10257, Lithuania; § VU LSC-EMBL Partnership for Genome Editing Technologies, Life Sciences Center, Vilnius University, Vilnius LT-10257, Lithuania

## Abstract

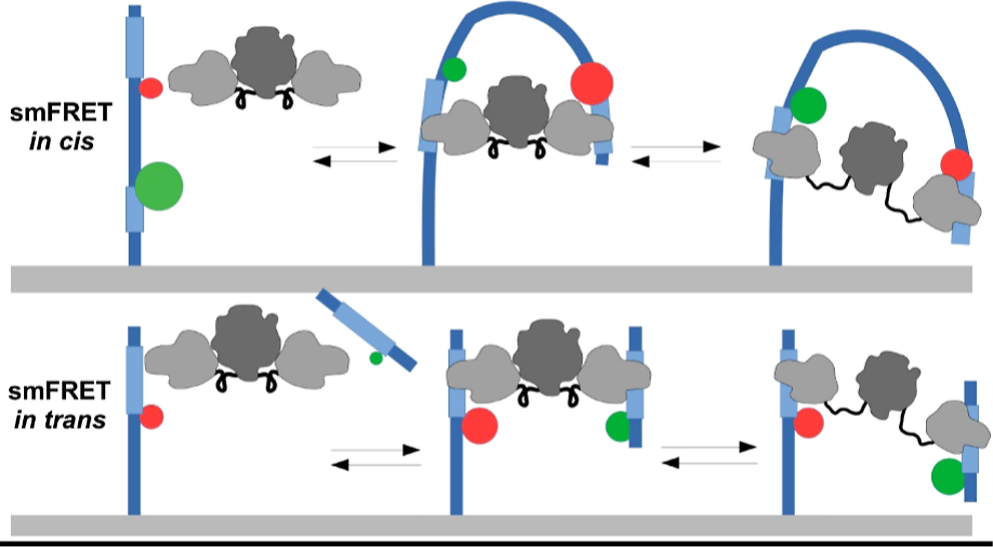

Protein–DNA interactions are fundamental to many biological processes. Proteins must find their target site on a DNA molecule to perform their function, and mechanisms for target search differ across proteins. Especially challenging phenomena to monitor and understand are transient binding events that occur across two DNA target sites, whether occurring in cis or trans. Type IIS restriction endonucleases rely on such interactions. They play a crucial role in safeguarding bacteria against foreign DNA, including viral genetic material. BfiI, a type IIS restriction endonuclease, acts upon a specific asymmetric sequence, 5-ACTGGG-3, and precisely cuts both upper and lower DNA strands at fixed locations downstream of this sequence. Here, we present two single-molecule Förster resonance energy-transfer-based assays to study such interactions in a BfiI–DNA system. The first assay focuses on DNA looping, detecting both “Phi”- and “U”-shaped DNA looping events. The second assay only allows in trans BfiI–target DNA interactions, improving the specificity and reducing the limits on observation time. With total internal reflection fluorescence microscopy, we directly observe on- and off-target binding events and characterize BfiI binding events. Our results show that BfiI binds longer to target sites and that BfiI rarely changes conformations during binding. This newly developed assay could be employed for other DNA-interacting proteins that bind two targets and for the dsDNA substrate BfiI-PAINT, a useful strategy for DNA stretch assays and other super-resolution fluorescence microscopy studies.

## Introduction

DNA–protein interactions play a key role in many biological processes,^1,2^ occurring at nanometer lengths and millisecond-to-second timescales. One aspect unifying these interactions is that a protein must find its target site on a DNA molecule to perform its function: cleavage, modification, activation of other proteins, etc.^3^ Target search mechanisms differ across proteins but can be divided into several classes: 3D diffusion (i.e., jumping), sliding, hopping, and intersegmental transfer.^4,5^ They also differ based on the number of DNA target sites that a protein binds. Proteins with a single DNA target binding domain utilize mechanisms from one (or a combination) of the target search classes.^6−8^ However, proteins with two DNA binding domains may simultaneously interact with two targets on a single DNA molecule (i.e., in cis interaction) or on two separate molecules (i.e., in trans interaction) and with different mechanisms.^9^ Temporal interactions with two target sites in cis and in trans often play an important role in target search and are critical in many cellular mechanisms.^10−13^ For example, precise timing of two DNA target binding events is important for transposons and transposases (such as the prototypical Tn7 family) in finding safe sites for insertion, efficient spread of the element, and avoiding unregulated insertion events.^14−16^

Restriction endonucleases play a crucial role in safeguarding bacteria against the intrusion of foreign DNA, including viral genetic material, and can also rely on multiple DNA interactions. Their classification depends on structural features, mechanisms of action, and other properties. Among the various types, type II restriction endonucleases are notable for their ability to selectively cleave double-stranded DNA molecules with remarkable precision. This cleavage occurs in the presence of magnesium ions, either within or near their recognition sites. One intriguing exception is BfiI, a type IIS restriction endonuclease: BfiI acts upon a specific asymmetric sequence, 5-ACTGGG-3, and precisely cuts both the upper and lower DNA strands at fixed locations downstream of this sequence in the absence of metal ions.

To perform mechanistic studies of such double-stranded DNA binders, one can employ standard molecular biology bulk ensemble-level methods. However, typically they only report an average value and require synchronization of all molecules in ensemble at the beginning of the measurement. Alternatively, structural methods, such as X-ray crystallography or cryo-electron microscopy, are extremely precise and provide atom-level resolution, but they only provide snapshots of molecules at different stages of complex formation. Continuous real-time monitoring of dynamic protein–DNA interactions can be performed upon tethering molecules of interest through force spectroscopy, fluorescence, Förster resonance energy transfer (FRET), atomic force microscopy (AFM), nanopores, and DNA flow-stretch assays.^17^ Single-molecule FRET (smFRET) approaches work especially well for high-throughput studies of protein–DNA interactions^18^ including CRISPR-Cas,^19,20^ SSB protein,^21^ RecA,^22^ DNA polymerase,^23,24^ RNA polymerase,^25,26^ and other proteins.^27−29^ The smFRET experiments can employ different designs, including (A) both dyes of a FRET pair linked to a single DNA fragment,^30^ (B) one dye on the DNA and the other on the protein,^8^ and (C) both dyes on a single protein.^31^ Notably, these designs are mainly employed to study DNA–protein interactions occurring in cis, where both target sites are on a single surface-immobilized DNA, but not in trans, where sites span two DNAs.

**Figure 1:**
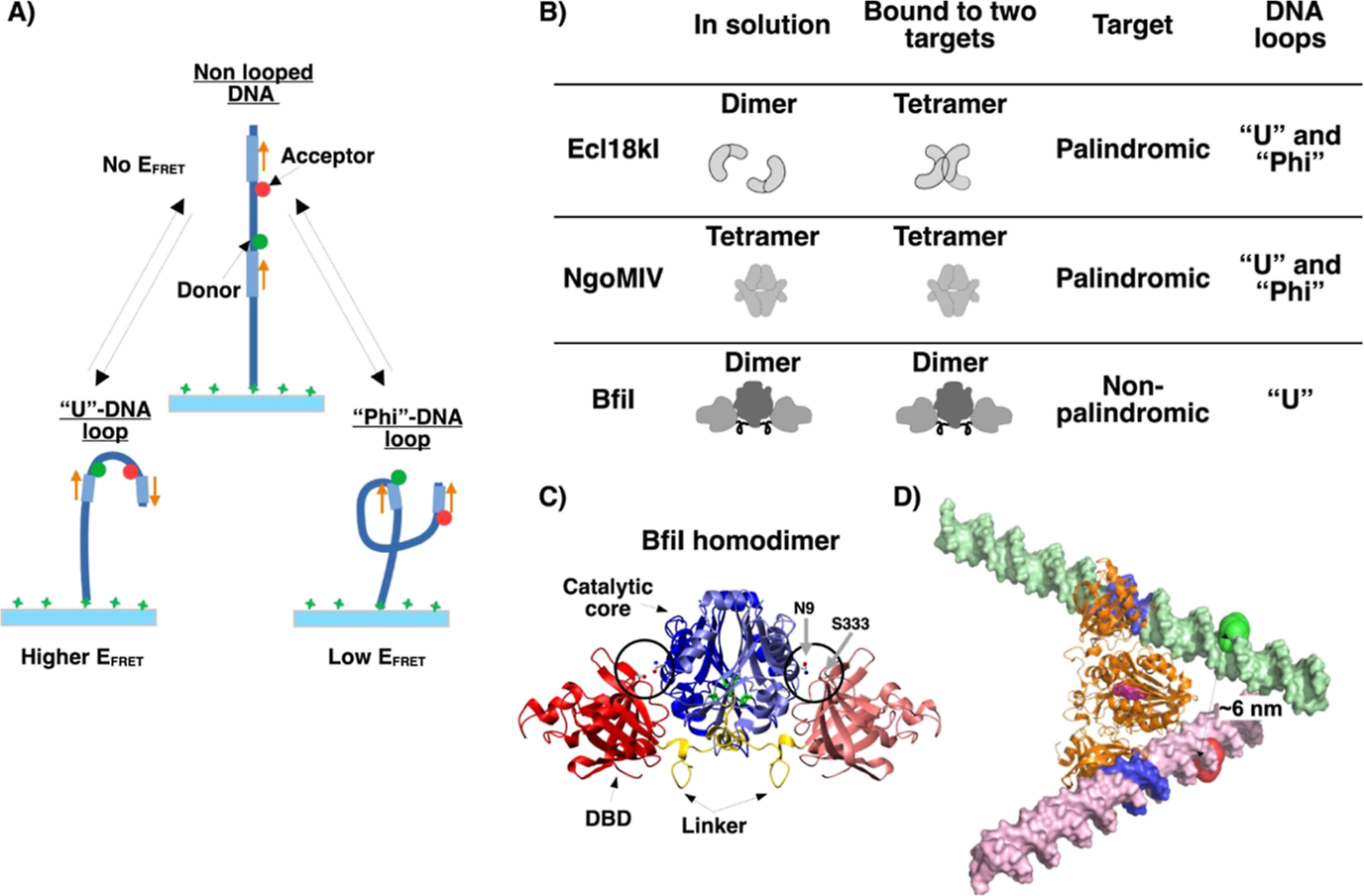
(**A)** Schematic diagram illustrating a single-molecule FRET-based DNA looping assay for probing in cis DNA–protein interactions across different restriction endonucleases. (**B)** List of our studied restriction endonucleases summarizing their interactions by DNA type, target (palindrome or not), and possible DNA loops. Ecl18kI–DNA interacts and produces “U”—(higher FRET efficiency—*E*_FRET_) and “Phi”-shaped (low *E*_FRET_) DNA loops.^32,33^ NgoMIV–DNA interact and form “U”—(high *E*_FRET_) and “Phi”-shaped (moderate *E*_FRET_) DNA loops.^34^ BfiI–DNA interact and form a “U”-shaped (low *E*_FRET_) DNA loop. (**C)** Overall BfiI homodimer structure without a DNA substrate (PDB ID: 2C1L).^35^ Each monomer is composed of the N-terminal PLD-like domain and the C-terminal DNA binding domain (DBD). Two N-terminal domains (light and dark blue) make a dimeric catalytic core flanked by two C-terminal DBDs (light and dark red). The linker connecting the N- and C-terminal domains is colored yellow. The active site residues are shown in green-colored balls and sticks. The K107A mutation makes BfiI catalytically inactive. The N9 and S333 residues (circled in the structure and indicated by the arrows) shown as balls and sticks were replaced with Cys to generate a double N9C/S333C mutant that allows introduction of a SS cross-link between the N- and C-terminal domain. Reproduced with permission.^36^ Copyright 2015, American Chemical Society. (**D)** Model of BfiI homodimer in complex with two double-stranded DNA molecules containing target sites based on the co-crystal structure of BfiI DBD with cognate DNA (PDB ID: 2C1L).^35^ Target sites on DNA molecules are marked in blue, the position of the donor fluorophore on DNA is marked by green-colored spheres, and the position of the acceptor fluorophore on DNA is marked by red-colored spheres. The active site of BfiI is marked by magenta-colored spheres.

We previously employed an smFRET approach leveraging DNA loops to study DNA binding by type IIF restriction endonucleases Ecl18kI and NgoMIV.^33^ Two Ecl18kI dimers bind two pseudo-palindromic DNA targets (5′-CCNGG-3′, N is any nucleotide) in cis, which leads to protein tetramerization and formation of either of two possible DNA loop types—“U”- or “Phi”-shaped (Figure 1A).^33^ In the “Phi”-shaped DNA loop, the conformation distance between FRET pair fluorophores was too high for probing by FRET methods and resulted in “no” FRET efficiency, much like non-looped DNA. By simultaneously monitoring the tethered fluorophore motion and FRET efficiency, we revealed the “Phi”-shaped loop and true non-looped DNA states.^32^ The homotetrameric REase NgoMIV^37,38^ can simultaneously bind two palindromic (5′-GCCGGC-3′) target sites and form both types of loops (Figure 1B).^34^ Both DNA loop shapes result in non-“no” FRET efficiency, except where high NgoMIV concentrations encourage different tetramers to bind a single DNA across its two target sites.

**Figure 2:**
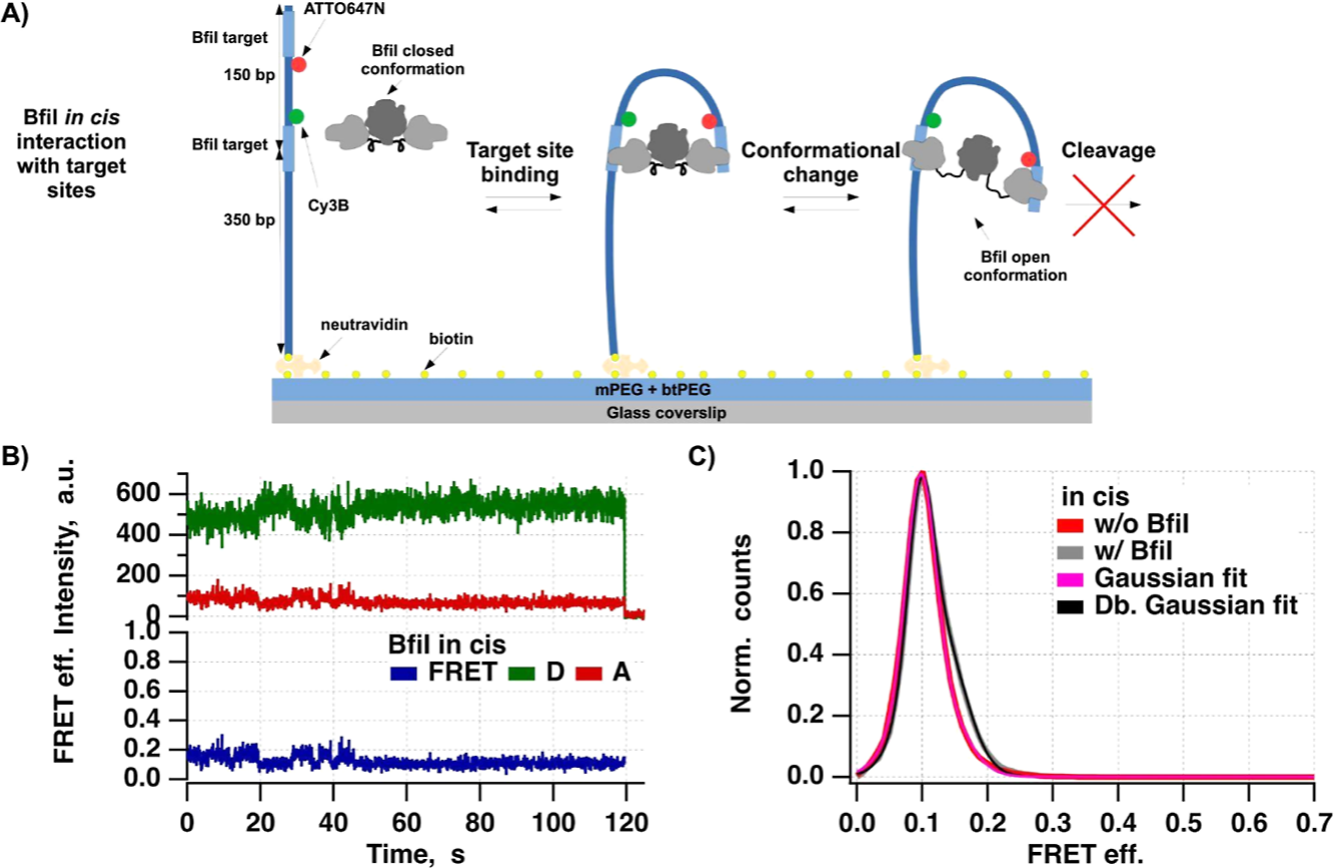
Results of the smFRET assay for BfiI–DNA in cis interaction studies. (**A)** Diagram illustrating the scheme of the smFRET-based DNA looping assay. Biotinylated double-stranded DNA fragments containing two BfiI target sites were immobilized on a glass surface via neutravidin. The target site located closer to the surface has the Cy3B fluorophore, and the surface distant site contains ATTO647N. Binding of BfiI protein to both target sites induces DNA looping, brings these two fluorophores in close proximity, increasing FRET efficiency. Subsequent conformational changes of BfiI between closed to open states may reduce the observed FRET efficiency. The active site mutation of BfiI makes it catalytically inactive and prevents target site cleavage. (**B)** Representative SM signals: donor intensity (green), acceptor intensity (red), and apparent FRET efficiency (blue), showing the DNA–BfiI interaction in cis. (**C)** Graph showing the distributions of FRET efficiencies of selected SM signals with (w/, number of molecules included: 315) and without (w/o, number of molecules included: 198) the BfiI protein. Distribution w/o BfiI was fitted by a single Gaussian function (fit values: center ∼0.1 ± 0.01 and width ∼0.03 ± 0.0004) and w/ BfiI—using a double-Gaussian function (fit values for the first peak: amplitude ∼0.52 ± 0.008, center ∼0.1 ± 0.0002, and width ∼0.03 ± 0.0003; fit values for the second peak: amplitude ∼0.52 ± 0.007, center ∼0.12 ± 0.0004, and width ∼0.06 ± 0.0003).

The previous approach had the weakness of formation of two different DNA loops and a DNA self-interaction. We have addressed these issues in this work and conducted protein–DNA interaction studies at the SM level with the type IIS REase BfiI either in the presence (probing in cis interaction) or in the absence of (probing in trans interaction) a DNA loop. Unlike Ecl18kI and NgoMIV, the BfiI homodimer contains separate domains for cleavage and recognition and is thus capable of binding in cis and in trans (Figure 2A). Binding of cognate DNA to BfiI presumably triggers a conformational change from the “closed” to “open” state, revealing an entrance to its active site.^35,36,39,40^ BfiI REase requires two copies of an asymmetric 5′-ACTGGG-3′ target site;^41,42^ therefore, it can only make a “U”-shaped DNA loop (Figure 1C,D). Since BfiI does not require Mg^2+^ ions for catalysis,^43^ we chose to study BifI protein with a K107A active site mutation^42^ (hereafter called the BfiI protein). We employed two different smFRET schemes to measure BfiI–DNA interactions, directly observing on-/off-target binding events in cis and in trans to characterize their durations and frequencies. We also describe and demonstrate a novel smFRET-based approach to study protein–DNA interactions in trans. Our work will be useful for studying additional DNA binding proteins such as transposon systems and their temporal interactions with two target sites either in cis or in trans, which play an important role in target search mechanisms and cellular functions.

## Materials and Methods

### Protein and DNA Fragments

The active site mutant BfiI was purified as described previously.^42^ The active site cross-linked mutant BfiI was prepared and purified as described previously.^36^ Protein concentrations are expressed in terms of dimers. The SDS–PAGE analysis of the cross-linked active site BfiI mutant (BfiI-SS) was performed as described previously.^36^ We synthesized 350 bp DNA fragments containing either one (for in trans interaction studies) or two (for in cis interaction studies) target sites for BfiI: 5′-ACTGGG-3′. The scheme for fragment synthesis was the same as described previously.^32^

The single-stranded DNAs were prepared by automated synthesis (IBA, Germany). First, the auxiliary DNA fragment for 150 bp inter-target distance was produced using pUC19 as a template and 5′-ACTGGGCTGTCTATTATTATTGCAGCAGCCACTGGTAAC-3′ and 5′-TGGGCTGCATTTTTATTATTGAGCGCAGATACCAAATAC-3′ as primers. A biotinylated fragment was produced from the pUC19 template using the non-modified 5′-TAGTGCACGCGGTGTTCGCTCCAAGCTGGGCTGTG-3′ primer and the 5′ terminally biotinylated 5′-biotin-CGTATGTCGTACCGGTAAGAACTCTGTAGCACCGCC-3′ primer.

DNA fragments for in cis interaction studies (i.e., DNA looping): The FRET pair-labeled fragment was produced using an auxiliary DNA fragment as the template and the following two primers: 5′-AGCGTAGC**ACTGGG**CTGTCTATTA**t**TATTGC-3′, where **t** is the internally ATTO647N (via base attachment)-modified-dT base, and 5′-GAGCACCGCGTGTAGC**ACTGGG**CTGCATTTAT**t**ATTATTG-3′, where **t** is the internally Cy3B (via base attachment)-modified-dT base. Next, the biotinylated and FRET pair-labeled fragments were digested by AdeI, ligated, purified, and stored at −20 °C until use.

DNA fragments for in trans interaction studies: A FRET pair-labeled DNA fragment containing a single BfiI target site was produced using an auxiliary fragment as a template and the following two primers: 5′-AGCGTAGC**ACTGGG**CTGTCTATTA**t**TATTGC-3′, where **t** is the internally ATTO647N (via base attachment)-modified-dT base, and 5′-GAGCACCGCGTGTAGC*CCTGGG*CTGCATTTAT**t**ATTATTG-3′, where **t** is the internally Cy3B (via base attachment)-modified-dT base. Next, the biotinylated and FRET pair-labeled fragments were digested by AdeI, ligated, purified, and stored at −20 °C until use.

The Cy3B-labeled dsDNA oligonucleotide (Cy3B-oligo) containing a BfiI target site was assembled by annealing oligonucleotides with the following sequences: 5′-GAGCACCGCGTGTAGC**ACTGGG**CTGCATTTAT**t**ATTATTG-3′, where **t** is the internally Cy3B (via base attachment)-modified-dT base, and 5′-CAATAATAATAAATGCAG**CCCAGT**GCTACACGCGGTGCTC-3′.

The Cy3B-labeled dsDNA oligonucleotide with no BfiI target site was assembled by annealing oligonucleotides with the following sequences: 5′-GAGCACCGCGTGTAGCCCTGGGCTGCATTTAT**t**ATTATTG-3′, where **t** is the internally Cy3B (via base attachment)-modified-dT base, and 5′-CAATAATAATAAATGCAG**CCCAGT**GCTACACGCGGTGCTC-3′.

### Sample Preparation

Each flow cell was assembled from a six-channel Sticky-Slide VI 0.4 (Ibidi, Germany) and a PEG-coated coverslip (Menzel Glaser, Braunschweig, Germany). The procedure of coverslip modification with PEG derivatives was as described previously.^44^ The flow cell channel was incubated with 0.5 mg/mL neutravidin (A-26666, Molecular Probes) in a yellow buffer (YB) [33 mM Tris-acetate (pH 7.9 at 20 °C), 66 mM K-acetate, and 1.5 mM BSA] for 3 min, washed with YB, incubated with ∼0.1 nM DNA in YB for 10 min, and then washed with YB. For the measurement of DNA–protein interactions, the cell was infused with 0.5 nM BfiI and 0.2 nM Cy3B-labeled dsDNA oligonucleotide in imaging buffer [IB, YB supplemented with 15 units/mL glucose oxidase (G6125, Sigma-Aldrich), 1% glucose (G0047, TCI Europe), 120 units/mL catalase (C9322, Sigma-Aldrich), and 2.5 mM UV-treated Trolox (Tx, 238813, Sigma-Aldrich)]. Each different condition reported in this work was measured in a separate flow cell channel.

### Single-Molecule Data Acquisition

An objective-type total internal reflection fluorescence (TIRF) microscope was used for single-molecule fluorescence movie acquisition.^44,45^ 532 and 635 nm lasers were set to ∼2 mW output, as measured after the objective and a ZT532/635rpc-XT dichroic mirror (Chroma; in the microscope filter turret). The fluorescence was filtered with a quadruple-band interference filter FF01-446/510/581/ 703 (Semrock), split by a T640lpxr-UF2 (Chroma), and imaged with an EMCCD (DU-897E-CS0-UVB, Andor) with 100 ms integration time.

### Data Analysis

In microscopy movies, fluorescent molecules were identified, and intensity versus time trajectories were extracted using a custom analysis package written in Igor Pro (Wavemetrics, Portland, OR), as previously described.^32,46^ Time trajectories were screened to exhibit characteristic single-molecule FRET features: clear donor–acceptor anticorrelated intensity changes, a typical single-molecule fluorescence intensity, and a single bleaching step. Protein binding events were detected in the intensity versus time trajectories automatically and then examined manually according to the following criteria: 532 nm donor excitation >30 a.u. and binding event length >5 frames.

## Results and Discussion

### SM Assay for the BfiI–DNA in cis Interaction

We began by investigating the ability of BfiI to bind two target sites in cis via our smFRET-based DNA looping assay.^32^ In our first scheme, we monitored in cis interactions and FRET efficiency changes within two target sites on a single surface-immobilized DNA fragment upon BfiI-induced DNA looping (Figure 2A).

First, we immobilized biotinylated dsDNA molecules containing two BfiI target sites on a glass coverslip surface via neutravidin. The first target site of the DNA molecule was labeled with a Cy3B fluorophore and the second target site with an ATTO647N fluorophore. In the presence of BfiI protein, we expected to observe two DNA states: looped and non-looped. The non-looped DNA state should result in the lowest possible apparent FRET efficiency, which we termed the “no” FRET level (in our system, it is higher than 0 FRET efficiency due to the cross-talk of spectral channels and acceptor excitation with the donor laser), since the fluorophores should maintain the greatest distance from one another (on average). Since BfiI can bind its non-palindromic recognition sites in only one orientation, DNA should adopt a “U”-shaped conformation upon protein-induced looping, causing an increase in FRET efficiency. After binding both target sites, the BfiI protein may undergo a conformational transition from “closed” to “open” state. Such conformational change should decrease FRET efficiency.

To measure the value of “no” FRET—apparent FRET efficiency in the non-looped state—we acquired a series of images of the DNA sample immobilized on the flow cell surface with and without the BfiI protein using a home-built TIRF setup (see Materials and methods) and 532 nm laser excitation. The most observed signals, whether with and without BfiI, showed characteristic SM behavior: strong donor signal intensity and single-step bleaching events (Figure 2B). FRET efficiencies from the extracted SM signals without BfiI revealed a single Gaussian FRET efficiency distribution centered at ∼0.1 FRET efficiency. In the presence of BfiI, the FRET efficiency distribution had an overlapping double-Gaussian shape. We attributed the first Gaussian to the non-looped DNA state because it coincided with the protein-free control (both centered at ∼0.1 FRET efficiency). The second Gaussian had ∼0.02 higher FRET efficiency, which we attributed to the looped DNA state (Figure 2C).

As predicted, we saw an increase in FRET efficiency in the presence of BfiI, which we interpreted as DNA loop formation due to binding of both targets. Therefore, in this assay, “no” FRET events could reflect several fundamentally different situations: (1) no protein bound to the DNA, (2) single-target site-bound BfiI, (3) two-target site-bound BfiI in the “open” conformation, (4) and BfiI–DNA interactions outside the target site. Meanwhile, non “no” FRET events could reflect a range of BfiI conformations. Due to the ambiguity of “no” FRET events, we cannot rule out the presence of other experimental states.

### **SM Assay for the BfiI–DNA Interaction** in trans

**Figure 3:**
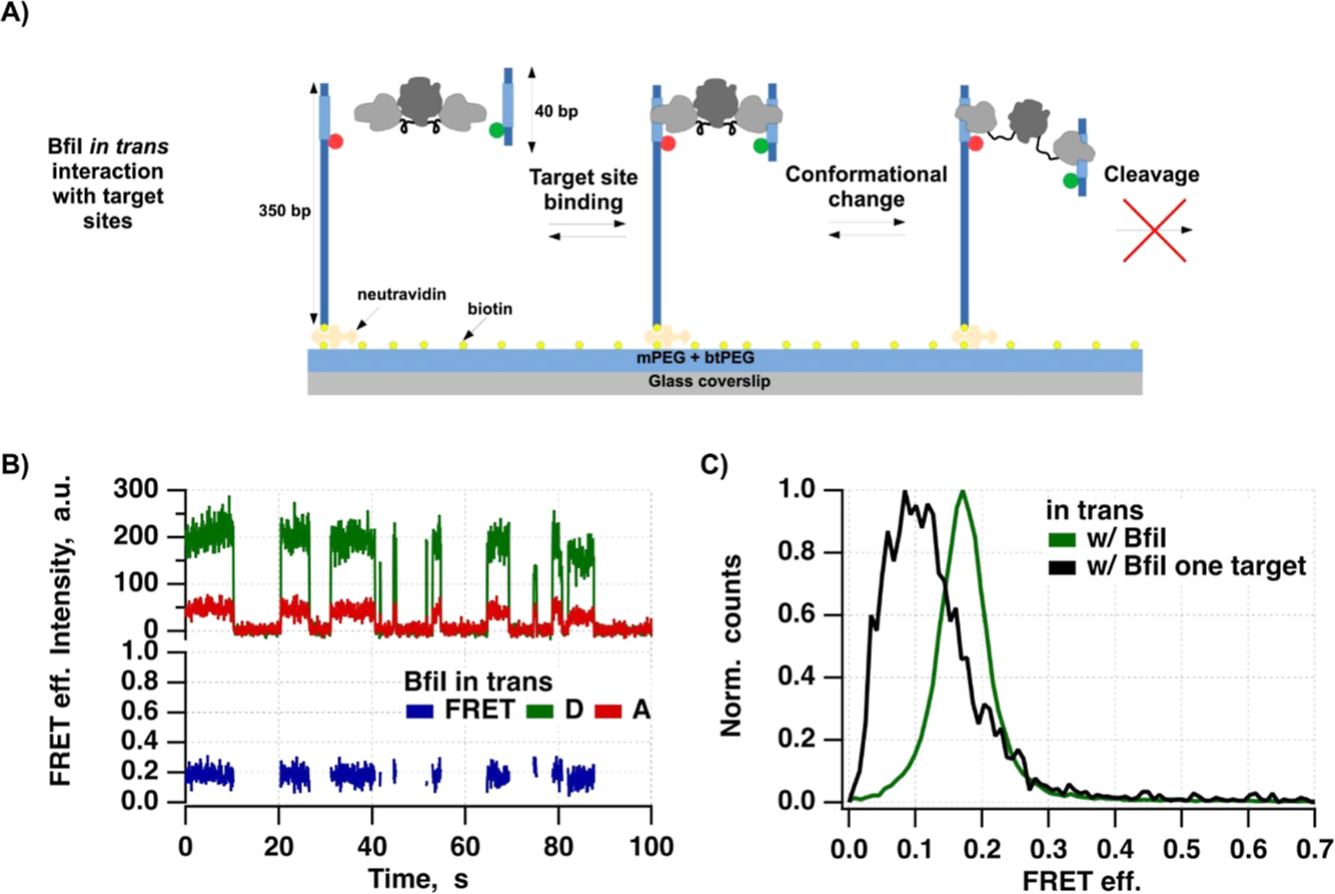
Results of smFRET BfiI–DNA in trans interaction studies. (**A)** Diagram illustrating the smFRET assay for the BfiI–DNA in trans interaction. Biotinylated double-stranded DNA (bt-dsDNA) fragments containing the BfiI target site and the FRET acceptor (ATTO647N) were immobilized on a glass surface via neutravidin. BfiI and the FRET donor-labeled dsDNA oligonucleotide (Cy3B-oligo) were present in the solution. BfiI can either first bind the Cy3B-oligo or the immobilized bt-dsDNA. Binding of this complex to the non-target location of bt-dsDNA results in “no” FRET efficiency and high Cy3B fluorescence emission intensity, whereas binding to the target site results in increased FRET efficiency and lower Cy3B fluorescence emission intensity. Once BfiI is bound to the target, it potentially can change conformation from closed to open state. Such transition should change the FRET efficiency from high to low. (**B)** Representative SM signals: donor intensity (green), acceptor intensity (red), and apparent FRET efficiency (blue), showing DNA–BfiI interaction in trans. (**C)** Graph showing the distributions of FRET efficiencies of the detected binding events for conditions w/ BfiI–oligoCy3B containing both the target site on oligoCy3B and the target site on the immobilized DNA fragment (number of molecules and states included: 311 and 590) and w/ BfiI–oligoCy3B and only a single target site on the immobilized DNA fragment (number of molecules and states included: 113 and 147).

To reveal BfiI–DNA interactions occurring only on target sites within surface-immobilized DNA molecules and to eliminate the possibility of DNA looping, we restricted our assay to in trans interactions by only allowing asymmetric BfiI targeting across two separate dsDNA molecules: we employed surface-immobilized biotinylated dsDNA molecules containing a single BfiI target site and labeled them with ATTO647N (near the target site at the surface-distant end of the fragment) and Cy3B (near the middle of the DNA molecule). Co-localization of these two dyes allowed us to locate surface-immobilized dsDNA molecules more reliably than with a single fluorophore. Alongside the double-labeled dsDNA, we used short Cy3B-labeled dsDNA oligonucleotides (Cy3B-oligo), which contained the second BfiI target site. These short Cy3B-labeled oligonucleotides were preincubated in a test tube with BfiI prior to experiments to form BfiI–Cy3B-oligo complexes (Figure 3A and Supporting Information Figure S1).

We hypothesized that this modified assay would exclude DNA loops from detection and enable monitoring of binding events for longer periods since Cy3B labels bleach less frequently. In our previously developed smFRET-based DNA looping assay, donor and acceptor fluorophores were always present; therefore, donor bleaching limited the observation time. Here, each new BfiI binding event on surface-immobilized DNA would likely occur with a different Cy3B fluorophore, allowing us to monitor the protein–DNA interactions for longer periods.

In principle, BfiI can bind surface-immobilized DNA molecules in either an on- or an off-target manner. We expected that in this assay, on-target binding of BfiI–Cy3B-oligonucleotide complexes would give a higher than “no” FRET efficiency and moderate Cy3B fluorescence intensity (Figure 3A), while off-target binding (anywhere within the immobilized DNA) should result in high Cy3B fluorescence intensity and “no” FRET efficiency. Most critically, closed to open state conformational transitions of BfiI should result in a shift from high to low (or “no”) FRET efficiency. However, conformational changes of BfiI, occurring at off-target binding events, would not be manifested as changes in FRET efficiency.

We first immobilized biotinylated dsDNA molecules onto the surface of a flow cell channel via neutravidin (Supporting Information Figure S1A) and imaged them via TIRF microscopy (with 532 and 635 nm wavelength excitations separately). Signal analysis resulted in a mean “no” FRET level of ∼0.1 FRET efficiency (Supporting Information Figure S2).

We then bleached Cy3B labels on the DNA molecules (532 nm laser for ∼3 min) and acquired images with 532 and 635 nm excitations separately (Supporting Information Figure S1B). We observed complete Cy3B bleaching and no evidence of ATTO647N bleaching, which was expected given the stability of ATTO647N dye in an oxygenated environment and its low absorption at 532 nm excitation. Next, we injected a mixture of BfiI preincubated with Cy3B-oligo together with the Tx and OS system and performed imaging as mentioned before (Supporting Information Figure S1C). A BfiI/Cy3B-oligo ratio of 2.5:1 was chosen to maximize the fraction of BfiI molecules interacting with a single Cy3B-oligonucleotide, leaving the second DNA binding domain free to interact with the surface-immobilized DNA. Such an experimental scheme allowed us to perform long-lasting imaging and circumvent the donor bleaching because each different BfiI binding event is with a new donor fluorophore and the acceptor is not excited directly (Supporting Information Figure S3).

The extracted FRET donor and acceptor SM signals showed fluorescence bursts of various durations that had clearly higher than noise Cy3B fluorescence emission intensities and FRET efficiencies (Figure 3B). Examination of traces revealed that ∼20% of all signals represented BfiI binding events. To characterize these events, we performed semiautomated detection using the criteria described in the Materials and Methods section. Distributions of FRET efficiencies that were made from all points of all detected binding events showed a distinct peak centered at ∼0.18 FRET efficiency (Figure 3C). This conformation of BfiI likely represents the same structure as the small shoulder that appears at ∼0.12 FRET efficiency upon Bfi-induced DNA loop formation in cis assay (Figure 2C). This suggests that with our in cis assay, due to the DNA looping affected DNA bending at the fluorophore attachment points, we measured lower FRET efficiency of the formed DNA–BfiI complex.

To validate our findings, we used the Cy3B-labeled dsDNA oligonucleotide without the BfiI target site in otherwise identical experiments (Figure 3C and Supporting Information Figure S4). These experiments showed a 10-fold lower number of binding events, with their peak FRET efficiency centered at <0.15. This negative control confirmed that on-target binding events must involve two BfiI recognition sites and that off-target binding events are rare. Thus, we could easily isolate signal parts that reflected BfiI binding to the immobilized DNA and discriminate between off- and on-target site binding events even though they were closely spaced in the apparent FRET efficiency space.

Based on the model structure of BfiI in closed conformation with bound DNA fragments (Figure 1D), the distance between FRET pair dyes is ∼6.5 nm. We expected to measure ∼0.4 FRET efficiency (R_0_ for the Cy3B and ATTO647N pair is ∼6.2 nm)^47^ upon binding both targets when the BfiI protein is in the closed state. However, the measured FRET efficiency for BfiI in cis and in trans assays was ∼0.12 and ∼0.2, respectively. These measured values were lower than expected for this protein in the closed state. Thus, it is likely that BfiI, upon binding of both DNA targets, changes conformation into the open state rapidly, and we only observe it in the open state during these ∼0.2 FRET efficiency binding or looping events.

To observe BfiI locked in the closed state, we performed experiments with a catalytically inactive (K107A mutation) cross-linked version of the BfiI-SS protein,^36^ in which the DNA binding domains are linked to the cleavage domain through a short linker. The cross-linking performed on BfiI resulted in a mixture of (1) a fully cross-linked protein (∼70%), where both DNA binding domains were cross-linked to the cleavage domain, (2) partially cross-linked BfiI (∼15%), where either one of the two DNA binding domains were cross-linked to the cleavage domain, and (3) a non-cross-linked protein (∼15%) (Supporting Information Figure S5). Binding with fully cross-linked BfiI-SS can only happen in a closed BfiI conformation, while partially cross-linked and non-cross-linked BfiI can bind in partially closed (one DNA binding domain—opened, while the second—closed) and opened states, respectively. Our smFRET in trans assay results with the BfiI-SS protein showed stable (no apparent FRET change during individual binding events) binding events (Supporting Information Figure S6A,B) that exhibited several FRET efficiency levels: ∼0.2, ∼0.35, and ∼0.5 (Supporting Information Figure S6C). We attributed ∼0.2 peak to the non-cross-linked BfiI (matching the results presented in Figure 3), ∼0.35 to the partially cross-linked BfiI, and ∼0.5 to the fully cross-linked BfiI. Therefore, it is likely that the observed small increases in FRET efficiency from 0.1 to 0.12 (Figure 2) and from 0.1 to 0.18 (Figure 3) represent the DNA looping/binding events mediated by BfiI existing mainly in the open state, where the FRET pair is separated by > 8 nm distance.

### BfiI–DNA Interaction Dynamics

After we determined that BfiI was able to bind the target site in trans on the immobilized DNA, we aimed to characterize the interaction dynamics of BfiI upon DNA binding. The BfiI conformational transitions from “closed” to “open” state were not visible in our assay, and we mainly monitored BfiI binding events in the open state that did not exhibit any FRET efficiency changes.

**Figure 4:**
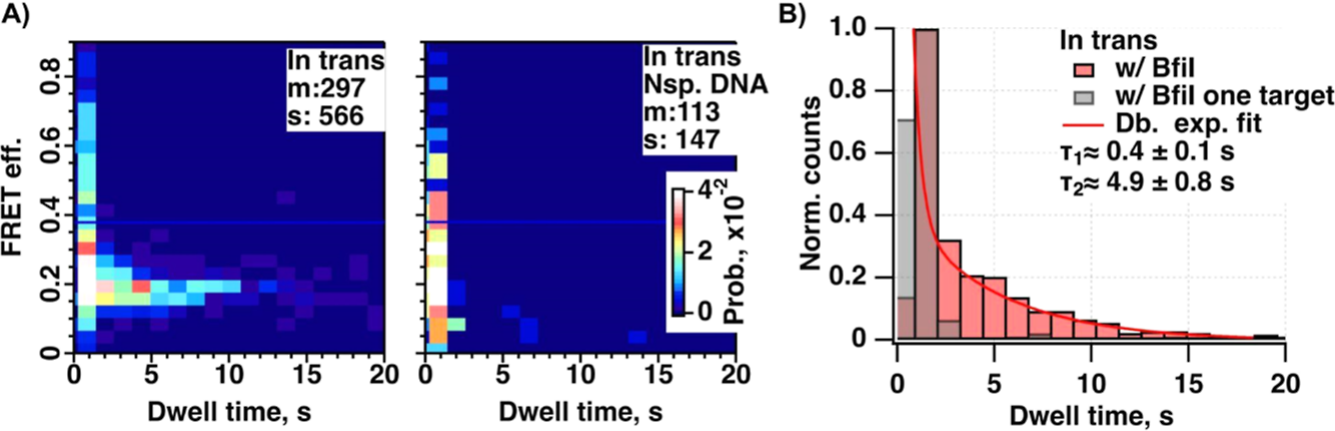
BfiI–DNA binding event characterization. (**A)** 2D histogram plot correlating the FRET efficiency of the binding event with its duration for conditions w/ BfiI–oligoCy3B containing the target site and w/ BfiI–oligoCy3B and only a single target site. Number of included molecules (m) and binding events (s) is indicated on top of each plot. (**B)** Horizontal line profiles for conditions w/ BfiI–oligoCy3B containing the target site (red) and w/ BfiI–oligoCy3B and only a single target site (gray) taken over the corresponding 2D histogram plots shown in panel **A**. The red-colored line profile was fit using a double-exponential function.

Stable FRET efficiency within a binding event allowed us to reliably calculate the mean FRET efficiency of a binding event, which would be less meaningful in the case of conformational changes within the binding event. To gain better understanding of binding events, we decided to correlate the average FRET efficiency (i.e., BfiI conformation) and duration (stability of the complex) of the binding event. For this purpose, we generated 2D histogram plots, where the *x*-axis represented the duration and the *y*-axis represented the average FRET efficiency of the event (Figure 4A). The plots visually demonstrated that in the presence of two target sites (both on the oligo DNA and on the immobilized DNA), binding events lasted longer than in the presence of a single target site (on the immobilized DNA molecule but not on the oligo in the solution).

The double-exponential function fitting to the binding events’ duration distribution showed a good fit (Figure 4B). The short component lasted for ∼0.4 s, and the long component lasted for ∼4.9 s. In comparison to BfiI binding events with two target sites, the observed binding events were notably shorter when only one target site was present and averaged at <0.5 s. The short binding events had variable FRET efficiencies since the durations were too short to reliably determine their average FRET efficiency. Such binding events were also observed with the non-specific DNA substrate, and therefore, they likely represent semispecific protein interactions with DNA molecules. The apparent long duration (characteristic dwell time 4.9 s) events represented the BfiI protein bound to two cognate target sites.

## Conclusions

Here, we demonstrated the application of two smFRET-based assays for studying simultaneous DNA interactions by a single protein, the BfiI endonuclease. With the smFRET-based DNA looping assay, we previously detected “Phi”- and “U”-shaped DNA looping events, as well as non-looped DNA events, in cis for Ecl18kI and NgoMIV REases. However, this approach could not discriminate between different types of BfiI protein binding events occurring on an immobilized DNA molecule. Also, we could not entirely rule out the possibility of different DNA loop conformations, which could potentially lead to different apparent FRET efficiencies of the looped DNA state. Besides, since both donor and acceptor fluorophores are always present, this smFRET-based DNA looping assay highly limits the meaningful observation time due to donor bleaching. In order to improve the encountered problems, we modified the previous assay to allow only in trans BfiI–target DNA interactions. This assay employed FRET acceptor-labeled surface-immobilized DNA and FRET donor-labeled dsDNA oligonucleotide, each containing a single binding target site. Dividing the BfiI target across two separate dsDNA molecules eliminated the possibility of DNA looping. This also allowed us to monitor the BfiI–target DNA interactions for longer durations since every new BfiI binding to the immobilized DNA event most likely happened with a new donor molecule.

Modified smFRET-based assay and TIRF microscopy allowed us to directly observe the BfiI–DNA binding events occurring on- and off-target. Our results showed that off-target binding events were short and lasted for <0.5 s, while the on-target events were longer and lasted for >4 s. We expected to detect BfiI conformational transitions from “closed” to “open” state upon cognate DNA binding by observing fluctuations of FRET efficiency from high to low. However, apparent FRET efficiency changes were not present. Therefore, after comparing the results with the cross-linked BfiI-SS (locked in the “closed” state), we concluded that the non-cross-linked BfiI in both of the employed assays binds target sites and rapidly transitions into the “opened” state.

The modified assay we describe here in a way is similar to protein-assisted PAINT experiments,^48^ used for single-stranded DNA labeling in super-resolution microscopy studies, whereas BfiI could be utilized for dynamic labeling of dsDNA substrates, in “BfiI-PAINT″ experiments. While the Argonaute protein, employed in the protein-assisted PAINT approach, can be loaded with a guide molecule of desired sequence (thus allowing more controlled labeling), the dsDNA substrate should contain at least one BfiI target sequence for successful labeling. We believe that this dsDNA labeling approach could prove useful for DNA Curtains experiments^44,49−52^ and other DNA stretch assays.^17,53^ Besides dsDNA labeling, it is likely that our developed assay will be useful for mechanistic studies of target search mechanisms and interaction dynamics of other important DNA binding proteins such as transposon systems or even artificially formed dimers of nucleases.

## Supporting Information Available

Scheme of smFRET assay for the BfiI–DNA in trans interaction; FRET efficiency distribution of the surface-immobilized, Cy3B, and ATTO647N-labeled biotinylated dsDNA before the Cy3B bleaching step; demonstration of the long-lasting imaging of the DNA–BfiI interaction in trans; DNA–BfiI interaction in trans negative control; SDS–PAGE analysis of the cross-linked active site BfiI mutant; and Cross-linked catalytically inactive BfiI in trans as a control for the closed state of BfiI (PDF)
