# Supplementary material for: smFRET Detection of Cis and Trans DNA Interactions by the BfiI Restriction Endonuclease
Source: J Phys Chem B. 2023 Jul 15;127(29):6470–8. doi: 10.1021/acs.jpcb.3c03269 (PMC10388346; doi:10.1021/acs.jpcb.3c03269)
Supplement: Supplementary file 1 — jp3c03269_si_001.pdf [file jp3c03269_si_001.pdf]

# Supporting information:

## smFRET detection of Cis and Trans DNA Interactions by the BfiI Restriction Endonculease

Šarūnė Ivanovaitė[‡§], Justė Paksaitė[§], Aurimas Kopūstas[§‡], Giedrė Karzaitė[‡], Danielis Rutkauskas[‡], Arunas Silanskas[§], Giedrius Sasnauskas[§], Mindaugas Zaremba[§], Stephen K. Jones Jr.[+], Marijonas Tutkus[‡§]*.

[‡] Department of Molecular Compound Physics, Center for Physical Sciences and Technology, Savanorių 231, Vilnius LT-02300, Lithuania.

[§]Vilnius University, Life Sciences Center, Institute of Biotechnology, Saulėtekio av. 7, LT-10257, Vilnius, Lithuania.

[+]VU LSC-EMBL Partnership for Genome Editing Technologies, Life Sciences Center, Vilnius University, Vilnius, Lithuania

*e-mail: marijonas.tutkus@gmc.vu.lt

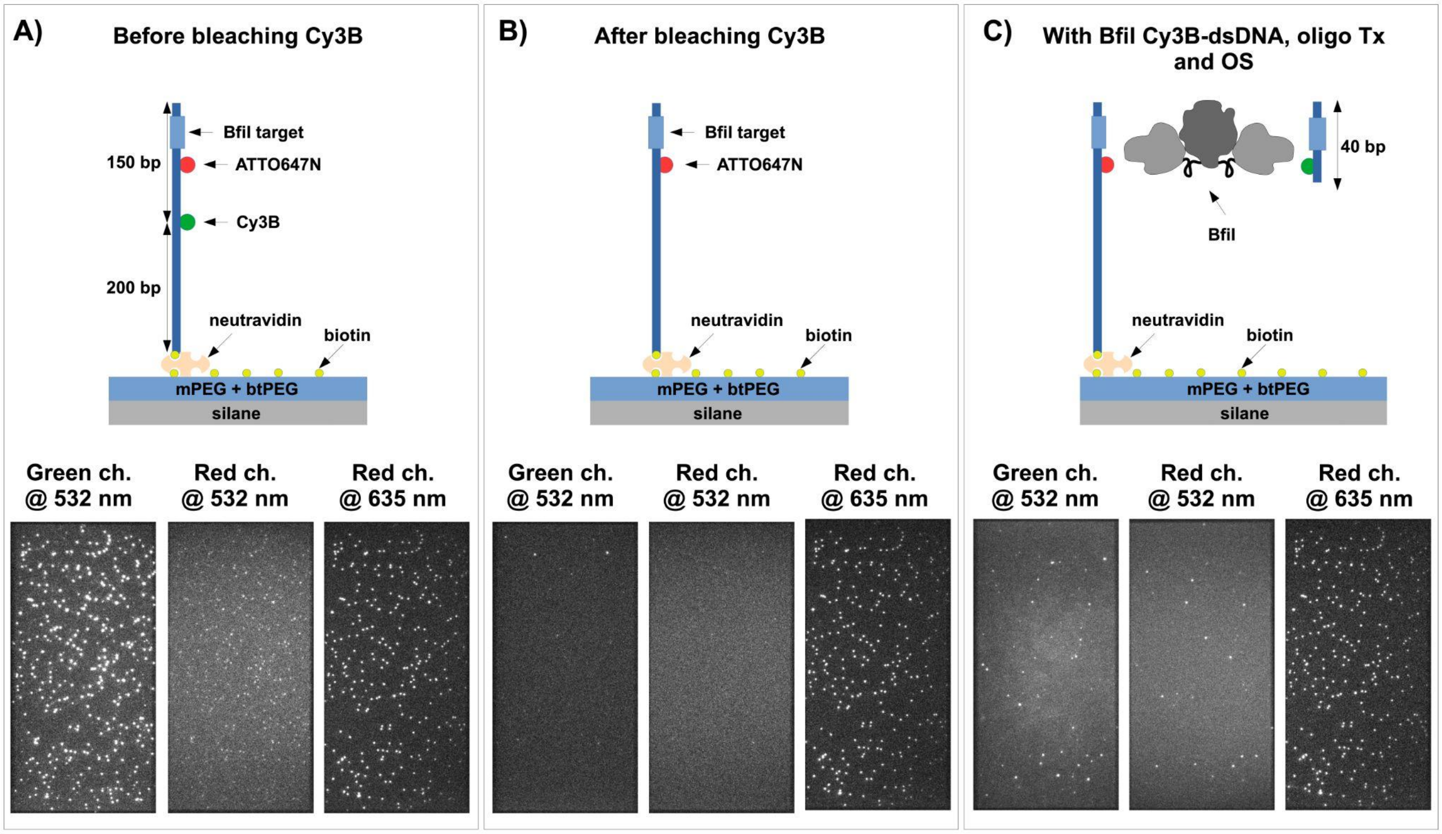


**SI Figure 1:** Diagrams illustrating the scheme of single-molecule FRET (smFRET) assay for BfiI-DNA in Trans interaction. **A)** Cy3B and ATTO647N labeled biotinylated dsDNA containing a single BfiI target site was immobilized on a PEGylated glass coverslip surface *via* neutravidin. The TIRF images acquired under 532 and 635 nm excitations showed co-localizing fluorescent spots. **B)** Cy3B on the biotinylated dsDNA was bleached out by shining 532 nm laser for 3 min. The TIRF images acquired under 532 and 635 nm excitations showed completely bleached Cy3B and no evidence of ATTO647N bleaching. **C)** Binding of BfiI was monitored by injection of BfiI and Cy3B-labeled double-stranded DNA oligonucleotide (Cy3B-oligo) in presence of Trolox (Tx) and oxygen scavenger (OS) system. The TIRF images acquired under 532 and 635 nm excitations showed increased background of Cy3B fluorescence and some fraction of co-localizing Cy3B and ATTO647N fluorescent spots.

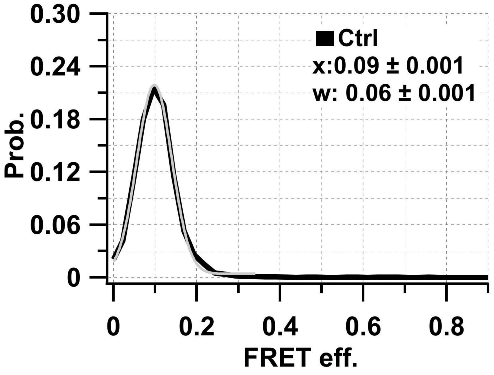


**SI Figure 2.** FRET efficiency distribution of Cy3B and ATTO647N labeled biotinylated double-stranded DNA immobilized via neutravidin before bleaching step (black) fitted with Gaussian fit (grey). The peak center position (x) and its width (w) values obtained from this fitting are indicated on top of the plot together with their error values.  Number of included molecules into this plot: 220.

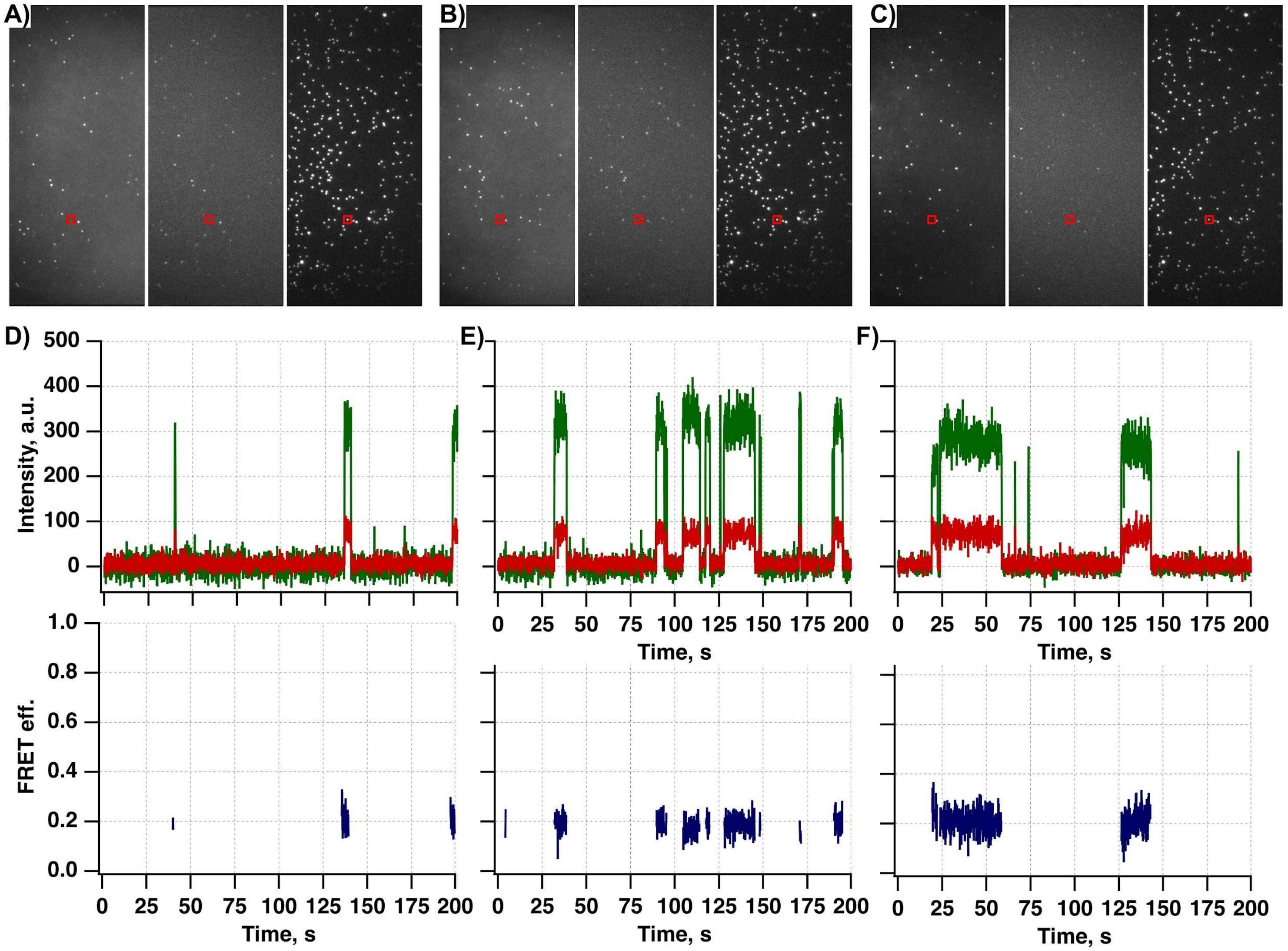


**SI Figure 3.** The long-lasting imaging of DNA-BfiI interaction In trans. 0.5 nM of BfiI and 0.2 nM of Cy3B-labeled dsDNA oligonucleotide with BfiI target site together with Tx and OS system incubated with the surface-immobilized single BfiI target containing DNA molecules. **A)** Illustrative single-molecule donor (left micrograph) and acceptor (middle micrograph) images that were acquired at 532 nm wavelength excitation and acceptor (right micrograph) image that was acquired at 635 nm wavelength excitation. **B)** The same surface position as in panel **A**, just after 200 s. **C)** The same surface position as in panel **B**, just after 200 s. **D-F)** Illustrative single-molecule donor (green) and acceptor (red) fluorescence intensity traces that were acquired under described conditions at 532 nm wavelength excitation along with the apparent FRET efficiency (blue) traces. These traces were extracted from the fluorescent spot indicated by red squares in panels **A**, **B**, **C**. Panel **D** corresponds to panel **A**, panel **E** corresponds to panel **B**, and panel **F** corresponds to panel **C**.

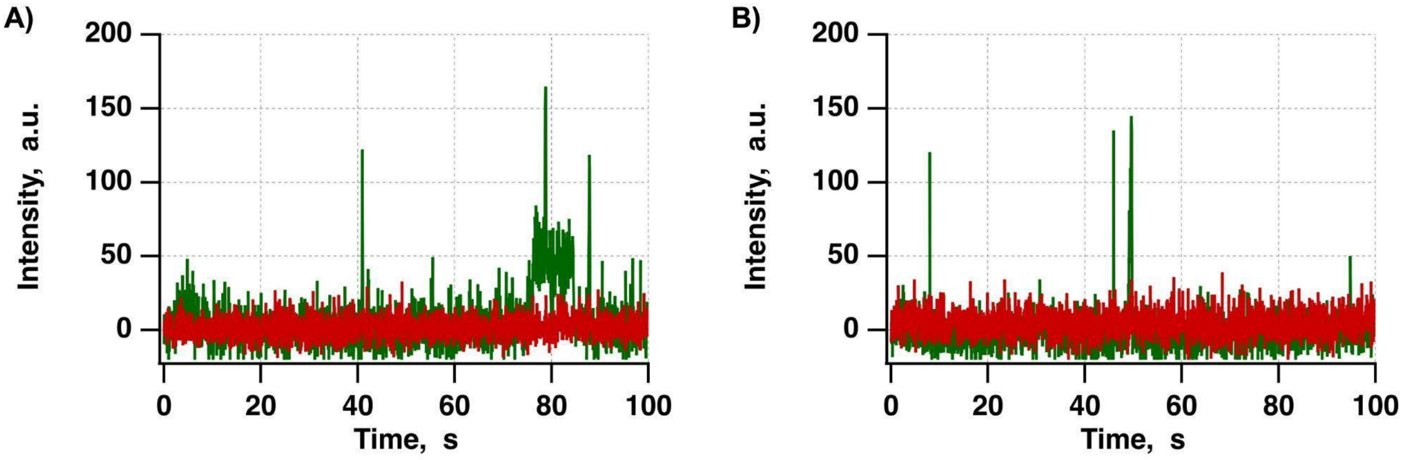


**SI Figure 4.** DNA-BfiI interaction *in trans* negative control. 0.5 nM of BfiI and 0.2 nM of Cy3B-labeled dsDNA oligonucleotide without BfiI target site together with Tx and OS system incubated with the surface-immobilized single BfiI target containing DNA molecules. A-B) Illustrative single-molecule donor (green) and acceptor (red) fluorescence intensity traces that were acquired under described conditions at 532 nm wavelength excitation.

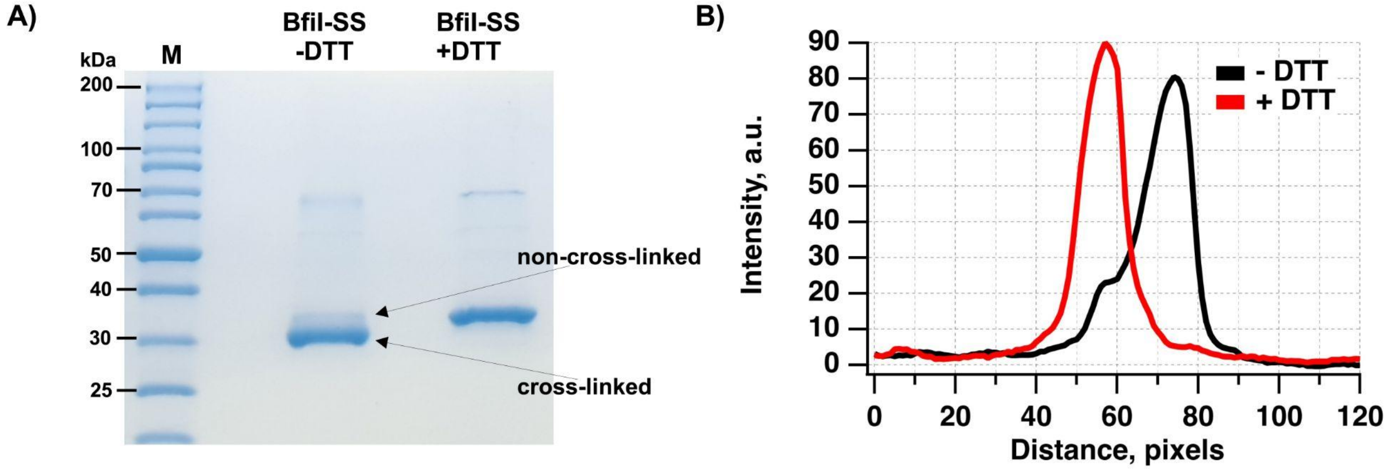


**SI Figure 5.** SDS−PAGE analysis of the cross-linked active site BfiI mutant (BfiI-SS). **A)** Protein samples of the BfiI-SS in the absence and presence of DTT were subjected to SDS−PAGE and visualized by Coomassie brilliant blue staining. Lane M contained molecular mass markers. **B)** Line-scans of SDS-PAGE lanes of Bfi-SS -DTT (black) and BfiI-SS +DTT (red). Line-scans illustrates that major fraction (>70 %) of BfiI-SS is crosslinked in the absence of DTT, and after reduction of the crosslinking bridge all BfiI-SS becomes non crosslinked. However, because the amount of non-crosslinked protein is lower than the cross-linked protein, most of the non-crosslinked protein will form heterodimer with the cross-linked protein.

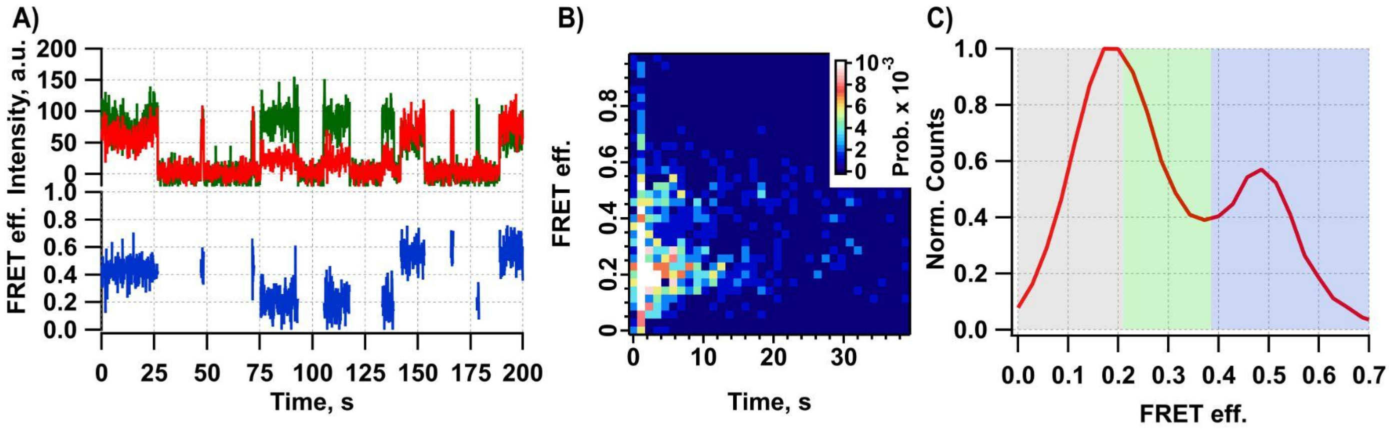


**SI Figure 6.** Crosslinked catalytically inactive BfiI (BfiI-SS) *in trans* as a control for closed-state of BfiI. 0.5 nM of BfiI-SS and 0.2 nM of Cy3B-labeled dsDNA oligonucleotide with BfiI target site together with Tx and OS system incubated with the surface-immobilized single BfiI target containing DNA molecules. **A)** Illustrative single-molecule donor (green) and acceptor (red) fluorescence intensity traces that were acquired under described conditions at 532 nm wavelength excitation. **B)** 2D histogram plot correlating FRET efficiency of binding event with its duration for conditions described in panel **A** (number of molecules, and states included: 302, and 1044). **C)** Graph showing the distributions of FRET efficiencies for all points of all detected binding events. Grey-filled area indicates the non-crosslinked population of BfiI, green-filled area indicates the partially-crosslinked population of BfiI, and blue-filled area indicates the crosslinked population of BfiI.
